# Risk of Benign Paroxysmal Positional Vertigo Modified by Diuretics—A Population‐Level Case‐Control Study

**DOI:** 10.1002/ohn.1282

**Published:** 2025-05-05

**Authors:** Marwin Li, Rebecca C. Chiffer, Hongyan Li

**Affiliations:** ^1^ Sidney Kimmel Medical College Thomas Jefferson University Philadelphia Pennsylvania USA; ^2^ Department of Otolaryngology Thomas Jefferson University Hospital Philadelphia Pennsylvania USA; ^3^ Department of Neurology University of Toledo College of Medicine and Life Sciences Toledo Ohio USA

**Keywords:** benign paroxysmal positional vertigo, carbonic anhydrase inhibitor, loop diuretic, thiazide diuretic

## Abstract

**Objective:**

This study aims to characterize diuretic use among patients with and without benign paroxysmal positional vertigo (BPPV) using a population‐level database.

**Study Design:**

A case‐control study.

**Setting:**

TriNetX US Collaborative Network.

**Methods:**

Subjects with ≥1 hospital visit between 2019 and 2024 were queried and stratified by age (18‐44, 45‐64, and 65+ years) and sex. Each cohort was then divided into those with/without BPPV. Patients with head trauma, middle/inner ear surgery, central vertigo, or migraine were excluded. The prevalence of diuretic use and vitamin D deficiency of each case cohort was compared against the control cohort of the same age/sex using Chi‐square analysis. This stratification and analysis were repeated for patients with a vestibular disorder, as well as those with/without Ménière's disease (MD).

**Results:**

Diuretic use was significantly more common in case cohorts than in control cohorts in the general population. In vestibular patients, thiazide and carbonic anhydrase inhibitor (CAI) use were more common in control cohorts, and loop use was less common. In MD patients, thiazide and loop use were more common in control cohorts, and CAI use did not differ significantly. In patients without MD, CAI use also did not differ, while thiazide and loop use were less common in control cohorts.

**Conclusion:**

All diuretics may alter the risk of BPPV. Their influences can be favorable or unfavorable, depending on the individual patient's medical history. Their effects might relate more directly to the efficacy of each diuretic class rather than their specific mechanisms of action.

Benign paroxysmal positional vertigo (BPPV) is one of the most common etiologies of vertigo in the general population. It is estimated to account for about 25% of all vertigo cases, as well as 60% of peripheral vertigo, and has a lifetime prevalence of 2.4%.^1^, ^2^ The pathophysiology of BPPV, while still being studied, is generally thought to be due to otoconia particles from the utricle and saccule that have detached and entered the semicircular canals. From there, these free‐floating particles are thought to either attach to the cupulae of the canals or remain suspended in the endolymph.^3^, ^4^ Either of these results in turbulence and the semicircular canals being inappropriately stimulated during head movements, causing a disturbing perception of movement. The factors responsible for displacing these particles, however, are frequently unclear.^5^

Studies have found that the risk of developing BPPV correlates with several disorders affecting calcium regulation, which may be attributed to the composition of the otoconia. Otoconia consist of a network of various proteins that form a scaffold upon which calcium carbonate binds to create a solid, mineralized structure.^6^, ^7^, ^8^, ^9^ These structures have been observed to degrade and fragment over time and therefore require maintenance by the local cellular environment.^10^, ^11^, ^12^, ^13^ Vitamin D deficiency has been strongly linked to BPPV, possibly by disrupting this calcium‐dependent maintenance.^14^, ^15^, ^16^, ^17^ Moreover, vitamin D supplementation has been associated with reduced BPPV recurrence.^18^, ^19^, ^20^ Other disorders involving calcium homeostasis, such as osteoporosis and hyperparathyroidism, have similarly been associated with BPPV.^15^, ^21^, ^22^, ^23^, ^24^ Overall, it seems that processes that decrease calcium availability or promote the breakdown of calcium structures may increase BPPV risk.

Due to this strong association with serum calcium, diuretic medications may theoretically affect BPPV risk as well. Diuretics are a large group of drugs that are commonly used to treat conditions such as hypertension and heart failure.^25^ Certain classes, such as thiazide and loop diuretics, are well‐known to affect serum calcium concentration.^26^ Thiazides are thought to promote the passive reabsorption of calcium from urine in the distal convoluted tubules of the nephrons, resulting in mild hypercalcemia.^26^, ^27^, ^28^ However, loop diuretics disrupt the electrochemical gradient of the thick ascending limb of nephrons that allows for calcium reabsorption, resulting in increased urinary loss of calcium and mild hypocalcemia.^25^, ^29^, ^30^ Other common classes of diuretics include carbonic anhydrase inhibitors (CAIs) and potassium‐sparing diuretics. Although the association is not as strong as with loop and thiazide diuretics, there is some recent evidence to suggest that CAIs may induce a mild hypocalcemia.^31^, ^32^, ^33^ Potassium‐sparing diuretics, however, do not seem to have a consistent, well‐documented effect on calcium metabolism independent of pre‐existing renal or endocrinologic pathologies.^34^, ^35^, ^36^, ^37^

Given these effects that diuretics have on serum calcium concentrations, it is possible that they may also extend to the cellular environment of the otoconia. If so, these effects may even modify BPPV risk. No such associations, however, have been reported in the literature, and there are currently no relevant clinical recommendations for reference, despite the high prevalence of this disease. Given that diuretics could theoretically have clinical implications in this regard, it is important to further explore this association so that diuretic prescribing practices may be adjusted accordingly. We hypothesized that the prevalence of BPPV may associate with the use of different classes of diuretics.

## Methods

The TriNetX US Collaborative Network was accessed on February 11, 2025, to use deidentified electronic health record (EHR) data encompassing ~117 million patients across 66 US Healthcare Organizations (HCOs). TriNetX‐LLC is a global federated live EHR research network and database. The Institutional Review Board at Thomas Jefferson University deemed this study exempt from review.

Subjects in this study must have at least 1 documented hospital visit between January 1, 2019, and January 1, 2024, at any of the 66 HCOs featured in the US Collaborative Network. Patients younger than 18 years at the time of visit or with a history of migraine, traumatic head injury, central vertigo, or middle or inner ear surgery were excluded. This yielded a total of 37,054,937 subjects from the general population, who were then stratified by age at the time of visit (18‐44 years, 45‐64 years, and ≥65 years), sex, vitamin D deficiency status, and diuretic use. For comparisons of diuretic use, only subjects without a history of vitamin D deficiency were included. Diuretic classes included in this study were thiazide diuretics (bendroflumethiazide, chlorothiazide, chlorthalidone, cyclothiazide, hydrochlorothiazide, hydroflumethiazide, indapamide, methyclothiazide, metolazone, polythiazide, and trichlormethiazide), loop diuretics (bumetanide, ethacrynate, furosemide, and torsemide), and CAIs (acetazolamide, brinzolamide, dichlorphenamide, dorzolamide, and methazolamide). Patients either used or did not use a particular class of diuretic before their visit.

**Figure 1:**
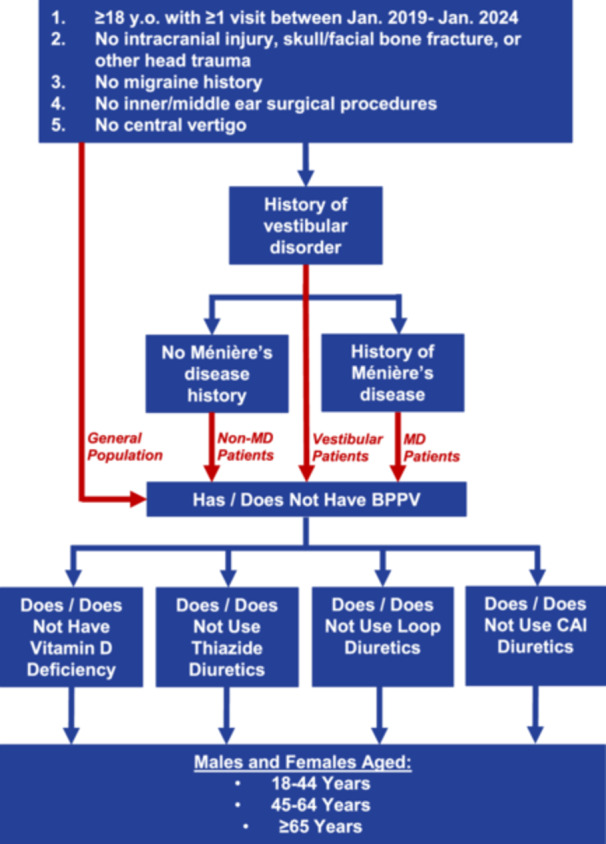
Hierarchy of criteria used for cohort generation for 4 different populations in the TriNetX US Collaborative Network database.

Three separate additional patient populations were then created using a similar query method as with the general population. Given that BPPV is often misdiagnosed or underdiagnosed in many clinical settings, there were concerns about a high false negative rate in the general population.^2^, ^38^, ^39^, ^40^, ^41^, ^42^, ^43^, ^44^, ^45^, ^46^ Therefore, one of these populations also required subjects to have a disorder of vestibular function, which encompassed BPPV and any non‐positional peripheral vestibular condition, who were diagnosed within the specified time period. This was done to analyze only those patients who were both properly evaluated for acute dizziness and had a non‐vestibular etiology ruled out, such as adverse drug effects or hypotension. We also recognized that Ménière's disease (MD), a relatively common non‐positional peripheral vestibular disorder for which diuretics are frequently prescribed, was a potential confounder.^47^ However, as MD often co‐occurs with BPPV, and may also frequently be misdiagnosed as BPPV or vice versa, MD was not excluded from this study.^48^, ^49^, ^50^, ^51^, ^52^ Rather, a population of vestibular patients with MD and a population of vestibular patients without MD were also queried. These 3 additional populations were each analyzed independently. Patient stratification is outlined in Figure 1. EHR codes used in this process are listed in Table 1.

**Table 1:** EHR Codes Utilized and Their Definitions

Code	Definition
	**ICD‐10‐CM**
E55	Vitamin D deficiency
G43	Migraine
H81	Disorders of vestibular function
H81.0	Ménière's disease
H81.1	Benign paroxysmal vertigo
H81.4	Vertigo of central origin
S02	Fracture of skull and facial bones
S06	Intracranial injury
S09	Other and unspecified injuries of head
	**CPT**
1010147	Surgical Procedures on the Middle Ear
1010223	Surgical Procedures on the Inner Ear
	**RxNorm**
	***Thiazides***
1369	Bendroflumethiazide
2396	Chlorothiazide
2409	Chlorthalidone
5487	Hydrochlorothiazide
5495	Hydroflumethiazide
5764	Indapamide
6860	Methyclothiazide
6916	Metolazone
8565	Polythiazide
10772	Trichlormethiazide
22033	Cyclothiazide
	***Loops***
1808	Bumetanide
4603	Furosemide
38413	Torsemide
62349	Ethacrynate
	***CAIs***
167	Acetazolamide
3353	Dichlorphenamide
6826	Methazolamide
60207	Dorzolamide
194881	Brinzolamide

A case‐control approach was taken for this study. Cases were the patient cohorts, after age and sex stratification, who were diagnosed with BPPV [BPPV(+)], whereas controls were those who do not have BPPV [BPPV(−)]. The proportion of each case cohort that used a particular diuretic drug class was compared against the proportion of the control cohort of similar age range, sex, and diuretic class. This was done using Chi‐square analysis, where *P* < .05 was statistically significant. Odds ratios were calculated with 95% confidence intervals (CI). A similar comparison between BPPV(+) and BPPV(−) cohorts was also conducted for prevalence of vitamin D deficiency, as well as for the use of each class of diuretic drug without age and sex stratifications. These statistical analyses were repeated for each of the 3 additional patient populations.

## Results

For the general population, a total of 37,054,937 subjects were queried in this study, of which 16,655,314 were male and 20,399,623 were female, with 164,780 BPPV cases and 36,890,157 controls. There were 14,864,834 patients who presented within the study window at the age of 18 to 44 years, 10,730,495 at 45 to 64 years, and 11,459,608 at 65+ years. Among the 34,439,762 total subjects without vitamin D deficiency, the overall prevalence of diuretic use was 7.96% for thiazide diuretics, 5.61% for loop diuretics, and 0.80% for CAIs. There were 325,879 total patients with a vestibular disorder, 37,978 with MD, and 288,128 with a non‐MD vestibular disorder. After excluding those with vitamin D deficiency, these totals became 257,384, 31,180, and 226,413, respectively.

### Vitamin D Deficiency and BPPV

For each patient population analyzed in this study, BPPV(+) patients overall had significantly greater odds of having concomitant vitamin D deficiency than their BPPV(−) counterparts. In the general population, BPPV(+) patients had 3.98 (95% CI: 3.94‐4.03, *P* < .0001) times greater odds for vitamin D deficiency than BPPV(−) patients after removing age and sex stratifications. For patients with vestibular disorders, these odds were 1.53 (1.49‐1.56, *P* < .0001) times greater. For patients with MD, odds were 1.60 (1.47‐1.74, *P* < .0001) times greater. For patients without MD, odds were 1.61 (1.57‐1.65, *P* < .0001) times greater.

### BPPV and Diuretic Use in the General Population

**Figure 2:**
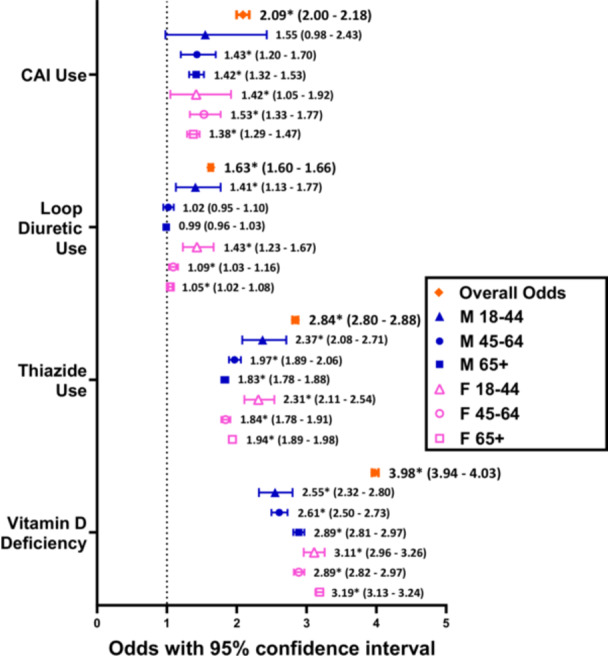
Odds ratios comparing the proportions of patients in the general population using a diuretic class in BPPV(+) cohorts versus age and sex‐matched BPPV(−) cohorts. “*” refers to statistically significant results with *P* < .05.

In the general population, BPPV(+) patients had significantly greater odds for using all 3 diuretic classes than BPPV(−) patients, at 2.84 (2.80‐2.88, *P* < .0001) times greater odds for thiazide use, 1.63 (1.60‐1.66, *P* < .0001) times greater for loop diuretic use, and 2.09 (2.00‐2.18, *P* < .0001) times greater for CAI use. This was consistently observed at nearly all levels of age and sex stratification, except for loop diuretic use in males aged 45 to 64 years (*P* = .5396) and 65+ years (*P* = .6280), and for CAI use in males aged 18 to 44 years (*P* = .0563) (Table 2 and Figure 2).

**Table 2:** Number and Percentage of Patients in the General Population in Each BPPV(+) and BPPV(−) Cohort Who Used a Particular Diuretic Class Before Hospital Visit

			*n* with diuretic use [% of cohort with diuretic use]		
Diuretic drug	Age (years)	Sex	BPPV(+)	BPPV(−)	*P* value
Use of thiazide diuretics	18‐44	Male	226 [3.81]	102,190 [1.64]	<.0001*
		Female	461 [3.74]	132,960 [1.65]	<.0001*
	45‐64	Male	2291 [16.35]	419,020 [9.02]	<.0001*
		Female	3692 [14.30]	430,629 [8.30]	<.0001*
	≥65	Male	6745 [25.51]	767,867 [15.74]	<.0001*
		Female	11493 [27.16]	862,336 [16.15]	<.0001*
	Total		24,908 [19.64]	2,715,002 [7.91]	<.0001*
Use of loop diuretics	18‐44	Male	78 [1.32]	58,143 [0.94]	.0024*
		Female	168 [1.36]	76,802 [0.95]	<.0001*
	45‐64	Male	748 [5.34]	242,717 [5.22]	.5396
		Female	1087 [4.21]	200,779 [3.87]	.007*
	≥65	Male	3774 [14.27]	701,320 [14.38]	.6280
		Female	5316 [12.56]	642,436 [12.03]	.0009*
	Total		11,171 [8.81]	1,922,197 [5.60]	<.0001*
Use of CAI diuretics	18‐44	Male	19 [0.32]	12,905 [0.21]	.0563
		Female	43 [0.35]	19,753 [0.25]	.0205*
	45‐64	Male	128 [0.91]	29,737 [0.64]	<.0001*
		Female	194 [0.75]	25,475 [0.49]	<.0001*
	≥65	Male	731 [2.76]	95,508 [1.96]	<.0001*
		Female	983 [2.32]	90,455 [1.69]	<.0001*
	Total		2098 [1.65]	273,833 [0.80]	<.0001*

* Statistically significant results with *P* < .05.

### BPPV and Diuretic Use in Patients With Vestibular Disorders

**Figure 3:**
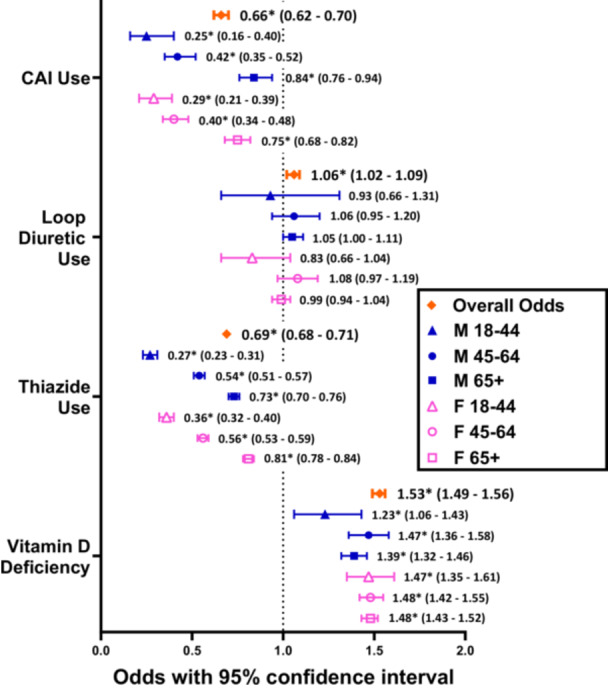
Odds ratios comparing the proportions of patients with a vestibular disorder using a diuretic class in BPPV(+) cohorts versus age and sex‐matched BPPV(−) cohorts. “*” refers to statistically significant results with *P* < .05.

Among subjects with vestibular disorders, BPPV(+) patients now had significantly lower overall odds of thiazide and CAI use, at 0.69 (0.68‐0.71, *P* < .0001) and 0.66 (0.62‐0.70, *P* < .0001) times the odds of their respective BPPV(−) cohorts. These associations were consistent at all levels of stratification. Overall loop diuretic use was slightly higher in BPPV(+) patients, with an OR of 1.06 (1.02‐1.09, *P* = .0006). However, no significant difference in loop diuretic use was observed at any level of stratification (Table 3 and Figure 3).

**Table 3:** Number and Percentage of Patients With a Vestibular Disorder in Each BPPV(+) and BPPV(−) Cohort Who Used a Particular Diuretic Class Before Hospital Visit

			*n* with diuretic use [% of cohort with diuretic use]		
Diuretic drug	Age (years)	Sex	BPPV(+)	BPPV(−)	*P* value
Use of thiazide diuretics	18‐44	Male	322 [3.59]	545 [12.18]	<.0001*
		Female	657 [3.51]	679 [9.27]	<.0001*
	45‐64	Male	3223 [15.62]	2491 [25.52]	<.0001*
		Female	5324 [14.12]	3133 [22.76]	<.0001*
	≥65	Male	9555 [24.87]	4797 [31.21]	<.0001*
		Female	16,338 [26.64]	6478 [31.00]	<.0001*
	Total		35,419 [19.06]	18,123 [25.31]	<.0001*
Use of loop diuretics	18‐44	Male	95 [1.06]	51 [1.14]	.6672
		Female	229 [1.22]	108 [1.47]	.1063
	45‐64	Male	969 [4.70]	432 [4.43]	.2923
		Female	1512 [4.01]	514 [3.73]	.1533
	≥65	Male	5127 [13.34]	1959 [12.75]	.0633
		Female	7189 [11.72]	2468 [11.81]	.7294
	Total		15,121 [8.14]	5532 [7.73]	.0006*
Use of CAI diuretics	18‐44	Male	27 [0.30]	53 [1.18]	<.0001*
		Female	75 [0.40]	101 [1.38]	<.0001*
	45‐64	Male	184 [0.89]	203 [2.08]	<.0001*
		Female	291 [0.77]	260 [1.89]	<.0001*
	≥65	Male	1059 [2.76]	500 [3.25]	.0019*
		Female	1404 [2.29]	637 [3.05]	<.0001*
	Total		3040 [1.64]	1754 [2.45]	<.0001*

* Statistically significant results with *P* < .05.

### BPPV and Diuretic Use in Patients With MD

**Figure 4:**
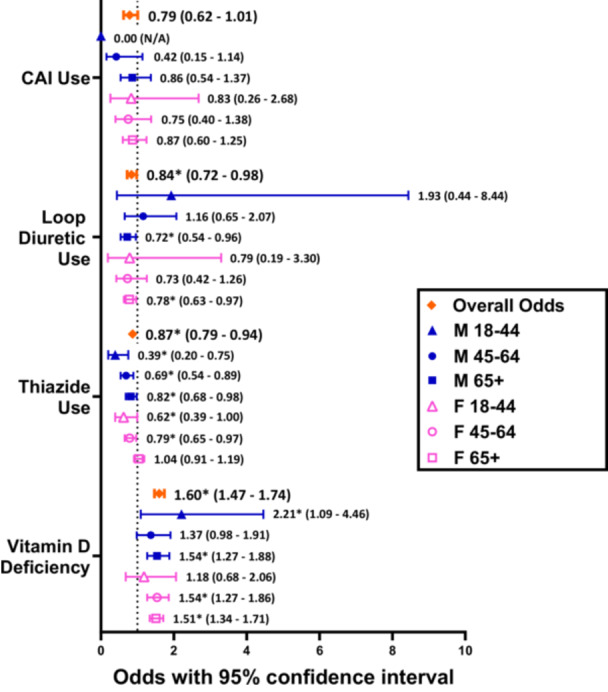
Odds ratios comparing the proportions of patients with MD using a diuretic class in BPPV(+) cohorts versus age and sex‐matched BPPV(−) cohorts. “*” refers to statistically significant results with *P* < .05.

Among subjects with MD, thiazide use was again significantly lower in BPPV(+) patients at nearly all age and sex stratification levels [except females aged 65+ (*P* = .5882)], with an overall OR of 0.87 (0.79‐0.94, *P* = .0010). However, CAI use was now similar between BPPV(+) and BPPV(−) patients, with an overall OR of 0.79 (0.62‐1.01, *P* = .0587) and no significant differences at any stratification level. Loop diuretic use was also significantly less common in BPPV(+) cohorts for the MD population, with an overall odds of 0.84 (0.72‐0.98, *P* = .0283) times that of BPPV(−) cohorts. When looking at the stratified analyses, this difference in loop diuretic use was only significant for males (*P* = .0256) and females (.0269) aged 65+ years (Table 4 and Figure 4).

**Table 4:** Number and Percentage of Patients With MD in Each BPPV(+) and BPPV(−) Cohort Who Used a Particular Diuretic Class Before Hospital Visit

Diuretic drug	Age (years)	Sex	*n* with diuretic use [% of cohort with diuretic use]		*P* value
			BPPV(+)	BPPV(−)	
Use of thiazide diuretics	18‐44	Male	11 [17.74]	420 [35.68]	.0038*
		Female	22 [18.97]	501 [27.33]	.0486*
	45‐64	Male	99 [34.74]	1742 [43.41]	.0043*
		Female	156 [33.91]	2166 [39.33]	.0220*
	≥65	Male	209 [38.14]	2936 [43.04]	.0255*
		Female	401 [42.39]	3904 [41.48]	.5882
	Total		898 [37.15]	11,669 [40.57]	.0010*
Use of loop diuretics	18‐44	Male	2 [3.23]	20 [1.70]	.3750
		Female	2 [1.72]	40 [2.18]	.7418
	45‐64	Male	13 [4.56]	159 [3.96]	.6179
		Female	14 [3.04]	227 [4.12]	.2590
	≥65	Male	54 [9.85]	899 [13.18]	.0256*
		Female	98 [10.36]	1211 [12.87]	.0269*
	Total		183 [7.57]	2556 [8.89]	.0283*
Use of CAI diuretics	18‐44	Male	0 [0.00]	30 [2.55]	.2032
		Female	3 [2.59]	57 [3.11]	.7516
	45‐64	Male	4 [1.40]	132 [3.29]	.0789
		Female	11 [2.39]	175 [3.18]	.3511
	≥65	Male	20 [3.65]	287 [4.21]	.5294
		Female	32 [3.38]	366 [3.89]	.4402
	Total		70 [2.90]	1047 [3.64]	.0587

* Statistically significant results with *P* < .05.

### BPPV and Diuretic Use in Patients With Non‐MD Peripheral Vestibular Disorders

**Figure 5:**
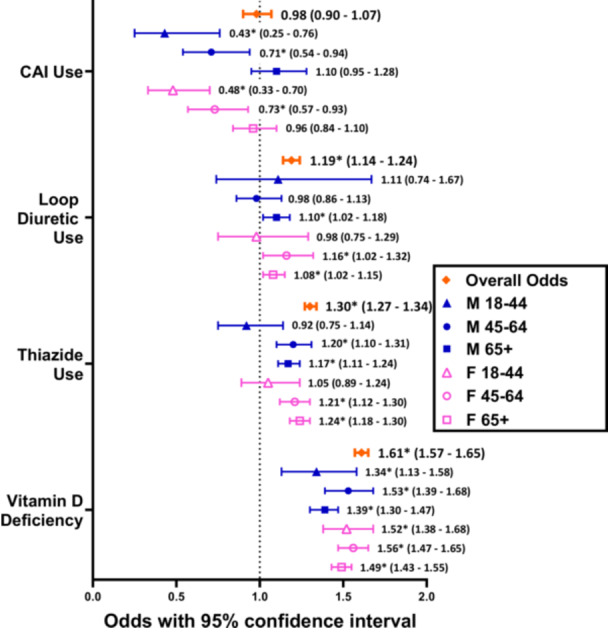
Odds ratios comparing the proportions of patients with a non‐MD peripheral vestibular disorder using a diuretic class in BPPV(+) cohorts versus age and sex‐matched BPPV(−) cohorts. “*” refers to statistically significant results with *P* < .05.

Among subjects with a non‐MD peripheral vestibular history, overall thiazide and loop diuretic use were significantly more common in BPPV(+) patients, at 1.30 (1.27‐1.34, *P* < .0001) and 1.19 (1.14‐1.24, *P* < .0001) times greater odds than their respective BPPV(−) cohorts. Overall CAI use was similar between BPPV(+) and BPPV(−) patients, with an OR of 0.98 (0.90‐1.07, *P* = .6632) times. For thiazide use, differences were not significant for males (*P* = .4436) and females (*P* = .5742) aged 18 to 44 years. For loop diuretic use, differences were only significant for females aged 45 to 64 years (*P* = .0209) and males (*P* = .0115) and females (*P* = .0137) aged 65+ years. For CAI use, differences were not significant for males (*P* = .1905) and females (*P* = .5669) aged 65+ years (Table 5 and Figure 5).

**Table 5:** Number and Percentage of Patients With a Non‐MD Peripheral Vestibular Disorder in Each BPPV(+) and BPPV(−) Cohort Who Used a Particular Diuretic Class Before Hospital Visit

Diuretic drug	Age (years)	Sex	*n* with diuretic use [% of cohort with diuretic use]		*P* value
			BPPV(+)	BPPV(−)	
Use of thiazide diuretics	18‐44	Male	312 [3.50]	125 [3.79]	.4436
		Female	635 [3.41]	179 [3.25]	.5742
	45‐64	Male	3121 [15.33]	755 [13.11]	<.0001
		Female	5172 [13.88]	974 [11.75]	<.0001*
	≥65	Male	9346 [24.67]	1868 [21.81]	<.0001*
		Female	15,947 [26.39]	2580 [22.41]	<.0001*
	Total		34,533 [18.82]	6481 [15.10]	<.0001*
Use of loop diuretics	18‐44	Male	93 [1.04]	31 [0.70]	.6130
		Female	226 [1.21]	68 [1.24]	.8931
	45‐64	Male	957 [4.70]	275 [4.78]	.8161
		Female	1498 [4.02]	288 [3.47]	.0209*
	≥65	Male	5071 [13.39]	1059 [12.36]	.0115*
		Female	7088 [11.73]	1258 [10.92]	.0137*
	Total		14,933 [8.14]	2979 [6.94]	<.0001*
Use of CAI diuretics	18‐44	Male	27 [0.30]	23 [0.70]	.0024*
		Female	72 [0.39]	44 [0.80]	<.0001*
	45‐64	Male	180 [0.88]	71 [1.23]	.0167*
		Female	280 [0.75]	85 [1.03]	.0113*
	≥65	Male	1038 [2.74]	213 [2.49]	.1905
		Female	1375 [2.28]	272 [2.36]	.5669
	Total		2972 [1.62]	708 [1.65]	.6632

* Statistically significant results with *P* < .05.

## Discussion

In the general population, BPPV(+) patients overall had significantly greater odds of having used all 3 classes of diuretics before diagnosis than BPPV(−) patients. These associations were consistent and significant at nearly all stratification levels for CAI and thiazide use, albeit were weaker for loop diuretics. For vestibular patients, thiazide and CAI use instead were significantly less common in BPPV(+) patients both overall and at all levels of stratification, whereas loop diuretic use was slightly more common, despite having no differences at each level of stratification. However, these associations differ when analyzing vestibular patients who do and do not have MD separately. MD BPPV(−) patients still demonstrate significantly greater odds of thiazide use but are also more likely to have used loop diuretics, and do not associate with CAI use. Meanwhile, non‐MD BPPV(−) patients had lower odds for thiazide and loop diuretic use but still did not associate with CAI use. As MD and non‐MD vestibular patients together should approximate the total vestibular disorder population in our study, the negative association of thiazide use with BPPV observed in all vestibular patients may have therefore been attributed to the MD population.

Overall, the evidence seems to demonstrate that diuretics do have an impact on BPPV risk, although this relationship may be nuanced by the patient's characteristics. In the general population, diuretic use was consistently greater among BPPV(+) patients, and could therefore be less preferrable for patients with other comorbidities that may predispose them to the development of positional vertigo. This is similar for non‐MD patients with peripheral vestibular disorders. However, for MD patients for whom diuretics are already indicated, the evidence most strongly suggests that thiazides may offer the greatest protective effect against BPPV. While loop diuretic use shared a similar association, this is unclear given the relatively low prevalence of their use in the study population.

The data provided by TriNetX are primarily intended for use in retrospective cohort population studies. Therefore, to help establish internal validity for this study, we calculated the prevalence of vitamin D deficiency among each patient population. Vitamin D deficiency was significantly more common among BPPV(+) patients than BPPV(−) patients for all populations in this study, at 1.53 to 3.98 times greater, which is very consistent with the existing literature.^14^, ^15^, ^16^, ^17^ It was also more common in older patient cohorts, as has been previously reported.^53^

To our knowledge, this is the first study that identifies a potential relationship between BPPV and diuretic use. As there are currently no pharmacologic practices recommended for the prevention of BPPV, these results could lead to the development of such strategies, particularly for patients with both hypertension and other BPPV risk factors. While our initial hypothesis attributed such a relationship to diuretics' effects on serum calcium, the varied associations in this study suggest that this explanation may be inadequate. It has been previously reported that increased water intake may correlate with reduced vertigo incidence, while dehydration may associate with increased BPPV incidence.^54^, ^55^, ^56^ Otoconia are thought to require constant cellular maintenance to prevent degeneration and fragmentation, with ischemic processes reportedly increasing BPPV risk possibly by interfering with the highly metabolic inner ear tissue.^10^, ^11^, ^12^, ^57^, ^58^, ^59^ Therefore, a low circulating intravascular volume, exacerbated by diuretic use, might increase overall BPPV risk due to poor perfusion of otoconia‐supporting tissues. This seems to explain the consistently higher odds of using all classes of diuretics seen in BPPV(+) patients in the general population. However, hypotension‐related vertigo cases misdiagnosed as BPPV in the BPPV(+) arm or missed BPPV diagnoses in the BPPV(−) arm could also create this association and cannot be excluded.

As thiazides and loops may induce greater diuretic effects than CAIs at therapeutic doses, dehydration may also explain why non‐MD BPPV(+) patients had higher odds of using thiazides and loops than non‐MD BPPV(−) patients.^60^, ^61^, ^62^ CAI use did not differ significantly between these 2 groups, possibly due to its generally milder diuretic effect and lower risk of compromising inner ear perfusion.

For patients with MD, an excess of endolymphatic fluid is thought to be present and diuretic use is often recommended.^47^, ^63^, ^64^ Also, because BPPV may present more frequently in MD patients, it has been theorized that hydropic changes may damage inner ear structures.^51^, ^65^ Therefore, diuresis may reduce BPPV risk in patients with MD, which the results of the MD population in this study seem to support. Thiazide and loop diuretics were more commonly used by non‐positional vertigo patients, while the milder CAIs again had no significant association. Total loop diuretic and CAI use was notably uncommon in this population; therefore, evidence for their associations is weaker than that for thiazide use.

Given the case‐control design of this study, these results cannot suggest cause and effect, but only the existence of an association. Other limitations include the dependence on third‐party reports of BPPV diagnoses to stratify patients. As this is a very commonly misdiagnosed condition, there is a possibility that the data set provided by TriNetX does not truly reflect the prevalence of BPPV in the study population.^38^, ^39^ Additionally, we could not obtain data regarding the duration of diuretic use by patients, nor their compliance with these medications, before their visits. For this reason, we were unable to determine whether the extent of diuretic use affected outcomes. TriNetX's built‐in analytic features are designed for retrospective cohort studies, and we did not possess access to individual patient data. Therefore, we were unable to perform propensity score matching when comparing diuretic use. To correct for this, we attempted to control external variables through exclusion criteria and addressed cohort size differences by including analyses without stratification. Future prospective studies are necessary to further clarify the effect that different diuretic classes may have on BPPV risk. These studies should also aim to require strict diuretic adherence and regular monitoring of subjects' volume statuses.

## Conclusion

This case‐control study used EHR data to identify statistically significant associations between use of certain classes of diuretic drugs and prevalence of BPPV at the population‐level. Overall, diuretic use appears to affect BPPV risk, but in a manner that is inconsistent with expected calcium changes. In the general population, thiazide, loop, and CAI diuretic use were each more common among BPPV(+) patients than BPPV(−) patients. Among MD patients, use of thiazide and loop diuretics was each significantly lower in BPPV(+) patients. However, among non‐MD patients, use of thiazide and loop diuretics was each significantly greater in BPPV(+) patients. These associations could be related to reduced perfusion of inner ear support tissues secondary to diuresis, which could affect otoconia maintenance and breakdown. Conversely, a certain degree of diuresis may lower BPPV risk in MD patients by reducing hydropic damage. The results most strongly suggest that diuretic use may, in general, increase the risk of BPPV in patients without MD and should be used with caution, especially for those with other comorbidities that predispose them to BPPV. However, patients with MD may have reduced BPPV risk while on thiazides than other classes. Further prospective investigation is required to properly characterize a dose–response relationship between diuretic use and BPPV outcomes, as well as to clarify the role that intravascular volume plays in this association.

## Author Contributions

**Marwin Li**, design, conduct, analysis, presentation; **Rebecca C. Chiffer**, analysis, review; **Hongyan Li**, design, conduct, analysis, review.

## Disclosures

### Competing interests

None.

### Funding source

None.
